# Impact of Humidity and Temperature on the Stability of the Optical Properties and Structure of MAPbI_3_, MA_0.7_FA_0.3_PbI_3_ and (FAPbI_3_)_0.95_(MAPbBr_3_)_0.05_ Perovskite Thin Films

**DOI:** 10.3390/ma14144054

**Published:** 2021-07-20

**Authors:** Marie Solange Tumusange, Biwas Subedi, Cong Chen, Maxwell M. Junda, Zhaoning Song, Yanfa Yan, Nikolas J. Podraza

**Affiliations:** Department of Physics & Astronomy and The Wright Center for Photovoltaics Innovation & Commercialization, University of Toledo, Toledo, OH 43606, USA; mariesolange.tumusange@rockets.utoledo.edu (M.S.T.); biwas.subedi@utoledo.edu (B.S.); cong.chen@utoledo.edu (C.C.); maxwell.junda@rockets.utoledo.edu (M.M.J.); zhaoning.song@utoledo.edu (Z.S.); yanfa.yan@utoledo.edu (Y.Y.)

**Keywords:** real time spectroscopic ellipsometry, perovskite thin films, optical properties, stability

## Abstract

In situ real-time spectroscopic ellipsometry (RTSE) measurements have been conducted on MAPbI_3_, MA_0.7_FA_0.3_PbI_3_, and (FAPbI_3_)_0.95_(MAPbBr_3_)_0.05_ perovskite thin films when exposed to different levels of relative humidity at given temperatures over time. Analysis of RTSE measurements track changes in the complex dielectric function spectra and structure, which indicate variations in stability influenced by the underlying material, preparation method, and perovskite composition. MAPbI_3_ and MA_0.7_FA_0.3_PbI_3_ films deposited on commercial fluorine-doped tin oxide coated glass are more stable than corresponding films deposited on soda lime glass directly. (FAPbI_3_)_0.95_(MAPbBr_3_)_0.05_ films on soda lime glass showed improved stability over the other compositions regardless of the substrate, and this is attributed to the preparation method as well as the final composition.

## 1. Introduction

Organic–inorganic metal halide-based *ABX*_3_ perovskites (*A* cation: methylammonium-MA, formamidinium-FA, cesium-Cs, rubidium-Rb; *B* cation: lead-Pb, tin-Sn; *X* anion: iodine-I, bromine-Br, chlorine-Cl) have gained tremendous attention in photovoltaic (PV) applications due to their high-power conversion efficiency which increased from 3.8% in 2009 to 25.5% [1,2,3] recently. Application of perovskite films to PV benefit from simple solution deposition processing, a tunable range of bandgap energies, and desirable optoelectronic properties, which include a high absorption coefficient above the bandgap energy, long carrier diffusion lengths, and long carrier lifetimes [4,5,6]. Solar cells made with these absorber layers are promising candidates for the next-generation high-efficiency PV technology [7]. The MAPbI_3_ perovskite is the pioneer among those used in solar cells and has been the most studied to date. Despite showing desirable optoelectronic properties, MAPbI_3_ degrades with exposure to humidity, oxygen, heat, and ultraviolet light [8,9,10,11]. Previous in situ real time spectroscopic ellipsometry (RTSE) studies conducted by Ghimire et al. on MAPbI_3_ upon atmospheric exposure have shown phase segregation into PbI_2_ and MAI starting at the interfaces of the film with the ambient and substrate [12]. It has been reported that MAPbI_3_ becomes hydrated under a humid environment in the dark and forms PbI_2_ with humidity exposure under illumination [13]. Another perovskite composition, FAPbI_3_, shows improved thermal stability compared to MAPbI_3_, however it may transform from the desired cubic, photo-conductive “black” perovskite α-phase into the “yellow” trigonal δ-phase in the presence of solvents and humidity [4]. Stability can be improved by tuning the cationic and anionic perovskite composition [14,15]. In particular, the *A* cation size is critical for the formation of a cubic perovskite structure [16]. When a mixture of MA and FA is used, the *A* cation size given by Goldschmidt tolerance factor falls in the 0.8–1.0 range, which is favorable for the cubic black phase perovskite structure to form, and the corresponding material stability is improved [17,18]. Recent studies indicate that partial Br substitution for I in MAPbI_3_ prevents ion migration in the perovskite and maintains favorable light absorption [19]. Substituting I with Br has also been reported to shrink the lattice parameter and increase the photogenerated-carrier lifetime and charge-carrier mobility [20]. All these studies provide general knowledge on improving the stability of organic–inorganic perovskites, however the level of stability for different perovskite film compositions in terms of complex optical response has been rarely explored.

Spectroscopic ellipsometry is a non-destructive, non-invasive measurement often used to explore the structural and optical properties of thin film materials. It measures changes in polarization state and amplitude of an incident light beam upon interaction with a sample either by reflection or transmission [21,22]. The complex optical properties and thicknesses of each component layer will impact this polarization state change via coherent multiple reflections. Data analysis of ellipsometric spectra is conducted by the construction of a parametric structural and optical model based upon a transfer matrix method from which physical properties, including the complex optical response and layer thicknesses, are extracted in a least squares regression fit to experimental ellipsometric spectra. In previous studies, thickness information gained from spectroscopic ellipsometry have been found to be in good agreement with that obtained from other techniques such as X-ray reflectivity, atomic force microscopy, scanning electron microscopy, and transmission electron microscopy [23,24,25,26]. When studying the dynamic evolution of properties of interest, in situ RTSE is used [12,27,28,29,30]. In RTSE, the sample is continuously measured so that the change in the polarization state of the incident light upon interaction with the sample is tracked as a function of time without physically moving the sample, although the sample characteristics may change.

Here in situ RTSE is used to track the variation of the optical and structural properties of MAPbI_3_, MA_0.7_FA_0.3_PbI_3_, and (FAPbI_3_)_0.95_(MAPbBr_3_)_0.05_ perovskite films deposited on soda lime glass and commercial fluorine-doped tin oxide (FTO) coated glass substrates under controlled relative humidity (RH) and temperature variations. These films are prepared using single-step spin coating and two-step solution processing [31,32,33]. The non-destructive and non-invasive nature of RTSE makes it the most suitable measurement method to track these changes. In addition, it enables the simultaneous determination of both optical and structural properties. Strong variations in optical and structural properties for MAPbI_3_ over time and the minimal variations for MA_0.7_FA_0.3_PbI_3_ and (FAPbI_3_)_0.95_(MAPbBr_3_)_0.05_ allow us to identify the factors influencing the stability of perovskites films. These factors include substrate type, mixing organic cations, incorporation of Br as a halide anion, and two-step versus single-step preparation methods. The RTSE data analysis methodology can be adopted in studying degradation of the perovskite absorber and other layers inside PV devices to assist in identifying stable materials for a large-scale industrial application.

## 2. Experimental Details

### 2.1. Perovskite Film Preparation

All perovskite films are prepared in a nitrogen (N_2_)-filled glove box to prevent exposure to ambient air. MAPbI_3_ and MA_0.7_FA_0.3_PbI_3_ films are prepared using single-step spin coating [31,32]. These films are deposited on soda lime glass and FTO coated glass (NSG Pilkington, Rossford, OH, USA TEC-15) substrates. PbI_2_ (TCI America, Portland, OR, USA), MAI (Greatcell Solar Materials, Queanbeyan, Australia), FAI (Greatcell Solar Materials, Queanbeyan, Australia), lead thiocyanate (Pb (SCN)_2_, Sigma–Aldrich, 99.5%), dimethyl sulfoxide (DMSO, Sigma–Aldrich, St. Louis, MO, USA 99.8%), and N,N-dimethylformamide (DMF, Sigma–Aldrich, St. Louis, MO, USA 99.8%) are used without further purification. The perovskite precursor solutions are prepared by dissolving mixtures of MAI, FAI, PbI_2_, Pb (SCN)_2_ in DMSO/DMF (*v*:*v* = 1:9), where the perovskite concentration is 1.5 M. The perovskite precursor solution is then spin-coated onto the substrate at 500 rpm for 3 s, and then at 4000 rpm for 60 s with diethyl ether dropped on the film at 10 s of the second step. The as-prepared perovskite films are annealed on a hotplate at 65 °C for 2 min and then at 100 °C for 5 min.

To prepare (FAPbI_3_)_0.95_(MAPbBr_3_)_0.05_, a two-step solution-processed preparation method is used as is described in [33]. Initially, 599.3 mg PbI_2_ is dissolved in a mixed solvent of 950 μL DMF and 50 μL DMSO. 70 mg FAI, 6 mg MABr, and 7 mg MACl are then dissolved in 1 mL isopropanol (IPA). To deposit the PbI_2_ film, 70 μL PbI_2_ solution is dropped onto substrate and spin-coated at 2000 rpm for 30 s, followed by annealing at 70 °C for 2 min. Then, the IPA solution is spin-coated on the as-prepared PbI_2_ film at 2000 rpm for 30 s to form the perovskite phase. Next, the samples are annealed at 150 °C for 15 min at 30–40% relative humidity in ambient air.

### 2.2. Real Time Spectroscopic Ellipsometry Data Collection and Analysis

**Figure 1:**
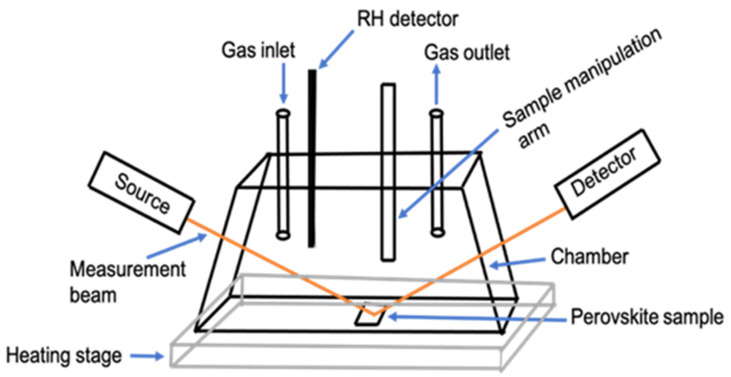
Schematic of the experimental setup for in situ real time spectroscopic ellipsometry (RTSE) data collection.

To transfer the prepared perovskite films for RTSE measurements, the films are loaded into a sealed measurement chamber (Figure 1) filled with nitrogen inside the glove box to prevent exposure to laboratory ambient air. The chamber containing the sample is placed on a temperature-controlled stage with a range from 7 to 70 °C (SCI-SCC6-L-F, Sciencetech Inc., London, ON, Canada). An infrared thermometer is used to confirm the temperature inside the chamber before and at the end of the measurements. Humidity is introduced by water vapor and nitrogen gas flows at a total rate of 5 standard cubic feet per hour (SCFH) into the chamber. 26% RH at temperatures of 7 and 70 °C, and 85% RH at 25 °C are measured within the chamber (Sensing solutions EVM GUI, Texas Instruments Inc., Dallas, TX, USA).

In situ RTSE measurements are performed to collect ellipsometric spectra in the form of *N* = *cos* (2*ψ*), *C* = *sin* (2*ψ*)*cos* (∆), and *S* = *sin* (2*ψ*)*sin* (∆) where the ellipsometric angles *ψ* and ∆ describe the relative amplitude and phase shift between the electric field components perpendicular and parallel to the plane of incidence [22]. Ellipsometric spectra (*N*, *C*, and *S*) are collected at an angle of incidence of 70° for 500 spectral points over a photon energy range from 1.25 to 6.00 eV using a single rotating compensator multichannel ellipsometer (M-2000FI, J.A. Woollam Co., Inc., Lincoln, NE, USA) [34]. Data is collected at 150 s intervals for each film to track changes in the complex optical response over time when the film is exposed to humidity and temperature. Selected time points are analyzed to track time dependent variations in the optical properties and film structure. Window effects of the chamber introducing an additional phase shift in the incident polarization state of the ellipsometer beam are accounted for through the use of Δ-offset parameters [35].

The experimental ellipsometric spectra are analyzed using a least square regression analysis in which the quality of fit between the model (*mod*) and the experimental (*exp*) ellipsometric spectra is defined in terms of the unweighted mean square error (*MSE*) [22]:

(1) $$MSE=1000\times \sqrt{\frac{1}{3n-m}\overset{n}{\underset{i=1}{\sum }}\left[{\left(N_{i}^{mod}-N_{i}^{exp}\right)}^{2}+{\left(C_{i}^{mod\;}-C_{i}^{exp}\right)}^{2}+{\left(S_{i}^{mod}-S_{i}^{exp}\right)}^{2}\right]}$$

where *n* is the number of measured values and *m* is the number of fit parameters.

To fit experimental ellipsometric spectra, a parameterized optical and structural model is used (CompleteEASE software, J.A. Woollam Co., Inc., Lincoln, NE, USA) to extract the optical response of the perovskite films in terms of complex dielectric function (*ε* = *ε*_1_ + *iε*_2_) spectra and structural parameters, which includes perovskite film thickness, the thickness of an interfacial layer forming between the substrate and perovskite film, and surface roughness layer thickness [36]. The full layer sequence consists of a substrate/perovskite + void interfacial layer/perovskite film/surface roughness/air ambient. The optical properties and the optical model used to describe soda lime glass and TEC-15 substrates have been reported in [37,38] respectively. Spectra in *ε* for the perovskite film are described using a parametric optical property model, which applies physically realistic parametric dispersion relations spanning from photon energies above to below the bandgap energy [39]. The mathematical equations for full parametric description of *ε* for these perovskites have been provided in [39,40]. The imaginary part of the dielectric function (*ε*_2_) is described by the sum of critical-point oscillators assuming parabolic bands (CPPB) above the direct bandgap energy and an Urbach tail below the bandgap [41,42]. The above-gap critical points (CPs) in these perovskite films are assumed to be excitonic [43]. The lowest energy CP in *ε*_2_ is considered to be the direct bandgap of the perovskite film. Spectra in *ε*_1_ is calculated as the sum of a Sellmeier expression, a constant additive term to the real part of *ε*_1_ denoted by *ε*_∞_, and Kramers–Kroning integration [39] of *ε*_2_ over this spectral range. Spectra in *ε* for the surface roughness layer and interfacial layer are represented by Bruggeman effective medium approximations [29] consisting of 0.5 void (*ε* = 1) and 0.5 bulk perovskite volume fractions.

## 3. Results and Discussion

**Figure 2:**
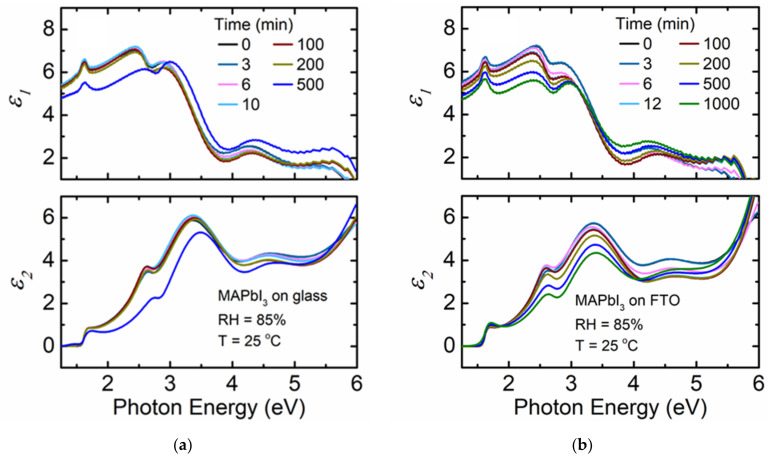
Complex dielectric function (*ε* = *ε*_1_ + *iε*_2_) spectra at selected time points for thin film MAPbI_3_ deposited on (**a**) soda lime glass and (**b**) fluorine-doped tin oxide (FTO) coated glass when exposed to 85% relative humidity (RH) at 25 °C. RH is introduced at 5 and 4 min for (**a**) and (**b**), respectively.

Figure 2a shows the variation in spectra in *ε* as a function of time for a MAPbI_3_ thin film deposited on a soda lime glass substrate when exposed to 85% RH at 25 °C. A noticeable decrease in the magnitude of *ε*_2_ is observed after 200 min of exposure of MAPbI_3_ to humidity. In Niu et al., MAPbI_3_ is exposed to 60% RH air at 35 °C, and the absorption feature in *ε*_2_ located at photon energies between the bandgap energy of 1.55 eV and 2.34 eV decreases sharply due to film degradation [44]. This interpretation provides context in understanding the decrease in magnitude of *ε* after 200 min of humidity exposure. Degradation throughout the MAPbI_3_ film is reflected in the change in optical properties represented on Figure 2a. It has been reported that the interaction between MA and water molecules leads to the desorption of MA, eventually degrading the surface of MAPbI_3_ by forming a hydrate phase MAPbI_3_·H_2_O [13,45]. Due to lack of stability of the hydrate phase, further decomposition into MAI and PbI_2_ could occur [45,46]. One of the possibilities to explain the observed variation in optical properties is that the film composition is no longer pure MAPbI_3_ when the film is exposed to humidity for prolonged times. Previous studies indicated hydrogen bonding between the oxygen in water and the hydrogen in the NH_3_ groups of MA cations [44,47]. Under exposure to humidity, the formation of that hydrogen bond will lead to an intermediate non-perovskite phase and this can cause the reduction in density of the perovskite film throughout the bulk [48]. It can be inferred that variation in optical properties can also result from the reduction in film density due to humidity induced film degradation.

**Figure 3:**
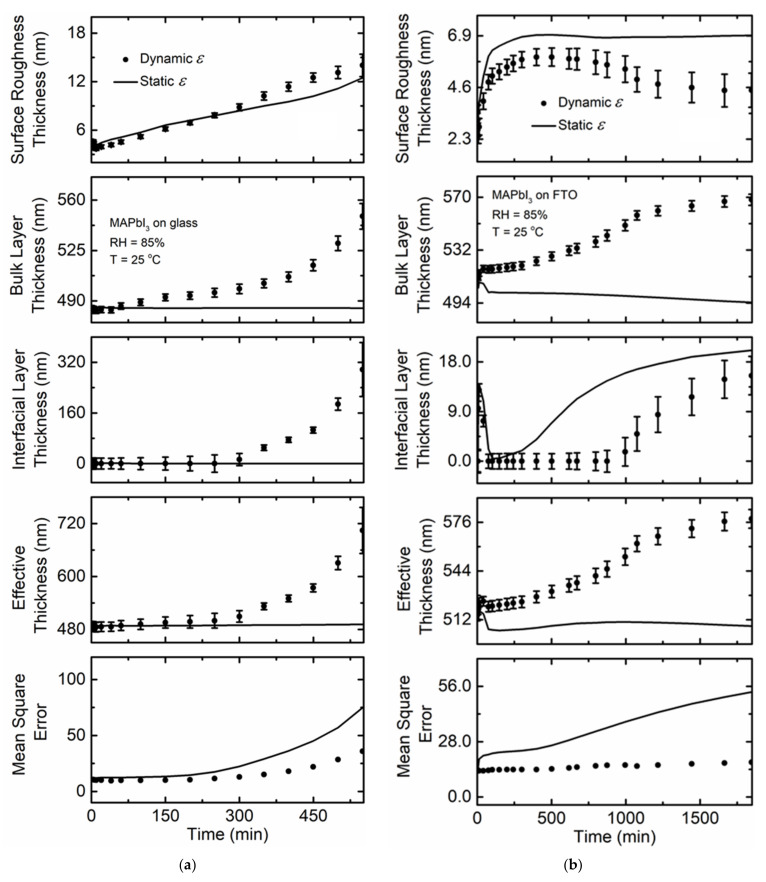
Surface roughness thickness, bulk layer thickness, interfacial layer thickness, effective perovskite thickness, and the quality of fit between the model and experimental ellipsometric spectra in the term of mean square error at selected time points as functions of time for thin film MAPbI_3_ on (**a**) soda lime glass and (**b**) FTO coated glass when exposed to 85% RH at 25 °C. Values obtained assuming the initial spectra in *ε* (time = 0 min; static) prior to exposure are represented by a solid line, and spectra in *ε* fit at each time point (dynamic) are represented by solid circles. RH is introduced at 5 and 4 min for (**a**) and (**b**), respectively.

Figure 3a shows the time dependence of the structural parameters in terms of the surface roughness, bulk layer, and interfacial layer thicknesses as well as the quality of fit between the model and the ellipsometric spectra represented by the *MSE*. Effective material thickness is calculated by combining the perovskite bulk film thicknesses with the interfacial layer and surface roughness layer thickness weighted with the perovskite material fraction in each layer respectively [12]:

(2) $$d_{effective,\;material}=\sum_{layers}f_{material\;\times }\;d_{layer}$$

where *d_layer_* is the thickness of each layer and *f_material_* is the fraction of perovskite material in each layer. This quantity is used to identify changes in the total amount of perovskite per unit area on the substrate. Two models are applied to analyze the RTSE data. One model applies spectra in *ε* obtained prior to humidity exposure to fit the experimental data throughout the measurement time. The other fits spectra in *ε* independently for each time point. As shown in Figure 2a, spectra in *ε* for MAPbI_3_ changes as a function of exposure time, therefore, the *MSE* obtained assuming static *ε* spectra obtained prior to humidity exposure is higher than that when spectra in *ε* are obtained at each time point. This difference in quality of fit indicates that both the structural parameters of the film and the intrinsic characteristics of the film reflected in *ε* vary with exposure to 85% RH.

Surface roughness thickness increases gradually as soon as humidity flow is initiated (Figure 3a). In the beginning, there is no optically detectable interfacial layer between the film and the glass substrate, however this lower density layer forms after 300 min of exposure. The evolution of this layer increases the effective film thickness, however changes in the surface roughness and bulk layer thicknesses also occur. Within 550 min of humidity exposure, the effective thickness has increased by 218 nm (Table 1). After 300 min, water molecules may have penetrated the film, potentially from the top surface along grain boundaries or laterally from the sample edges, to the interface between the film and the glass substrate. Due to change in spectra in *ε* representing the entire perovskite film, it is likely that water transport from both directions occurs simultaneously. The increase in effective thickness may be due to an increase in unit cell volume caused by the hydration of MAPbI_3,_ which will reduce the film density [49]. Considering the lattice volume of MAPbI_3_ and MAPbI_3_·H_2_O to be 247 Å^3^ and 263 Å^3^ respectively, conversion of MAPbI_3_ to MAPbI_3_·H_2_O causes a volumetric lattice expansion of 6% based on the relative lattice parameters of both phases [49,50]. This result indicates that water molecules intercalated into the perovskite may have increased the volume of the unit cell, which leads to the increase in the thickness of the film and a reduction in density. The increase in volume of the unit cells due to hydration alone is not sufficient to describe the observed increase in effective film thickness. Other factors, such as decomposition and phase segregation, indicated by the presence of the interfacial layer with the substrate may have contributed to the increase in effective thickness. It should be noted that the *MSE* increases steadily with time using both initial static spectra in *ε* or allowing *ε* to vary for each time point. As even the model assuming changes in *ε* over time possesses a substantially large *MSE* relate to other samples (Table 1), it is likely that water infiltration might initiate breakdown, particularly along the interface. In addition, PbI_2_ and MAI phase segregation might have occurred well before the final time point analyzed, which is consistent with the results reported by Ghimire et al. [12]. Briefly, we are not accurately capturing exactly how the film is changing and breaking down at long times since the *MSE* is quite high regardless of either the static or dynamic model used.

Figure 2b shows the variation of spectra in *ε* for a MAPbI_3_ film deposited on FTO coated glass substrate exposed to the same conditions. A decrease in the magnitude of spectra in *ε* occurs steadily with time. Smaller *MSE* values for MAPbI_3_ deposited on FTO coated glass compared to the film directly on soda lime glass implies that the film deposited on FTO does not degrade as much as that on bare glass. Even though the optical properties for the perovskite film on FTO coated glass visually change more during exposure time, the higher *MSE* for the film deposited on bare glass indicates that the structural and optical property model for the starting material is inadequate to describe MAPbI_3_ during the degradation process. We can assume that the intrinsic properties of the film on FTO change more, but perhaps these changes are a sign of large-scale stability since it does not break down to the extent of the film on bare glass. A comparative study conducted on MAPbI_3_:Cl perovskite films grown on glass, glass/FTO, and glass/FTO/TiO_2_ using photoluminescence indicated that films on FTO and on TiO_2_ show more stable behavior over that on glass as oxygen can diffuse through the surface from the layer below. The underlying surface roughness was found to be a key factor in the morphology of the perovskite grains and in the resulting concentration of defects by providing a different nucleation site density and impacting the dimensionality of the grain growth dynamics [51]. Minimal increase in effective thickness is observed in Figure 3b compared to that for MAPbI_3_ deposited on soda lime glass as listed in Table 1. The small variation in effective thickness for MAPbI_3_ deposited on FTO emphasizes that this film does not decompose or delaminate to the same extent as that of the same composition film deposited on glass. This behavior is also reflected in *ε* as the film on FTO appears more uniform after 1000 min of exposure compared to the film on glass, which shows more substantial changes up to 500 min. In the case of MAPbI_3_ deposited on glass, the huge increase in *MSE* suggests that the structural and optical property model applied no longer adequately describes this sample. The MAPbI_3_ film on glass is likely to have completely or partially degraded or slightly delaminates as the large change in effective thickness is driven by the increase in the interfacial layer (Figure 3a). The interfacial layer is nearly as thick as the film itself in that case.

**Figure 4:**
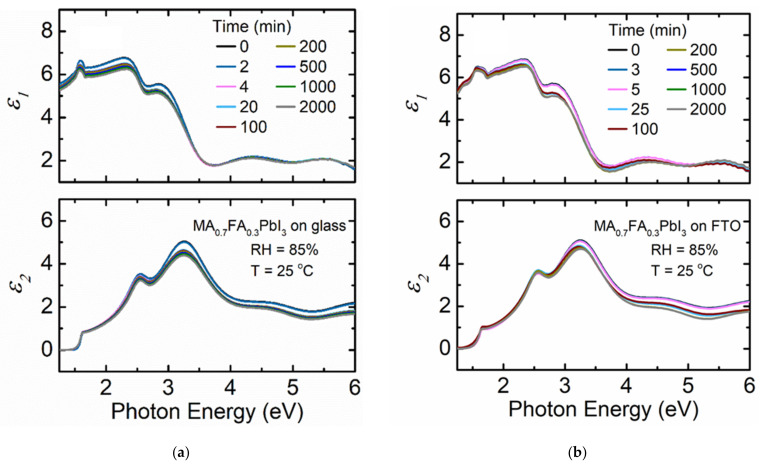
Spectra in *ε* at selected time points for thin film MA_0.7_FA_0.3_PbI_3_ deposited on (**a**) soda lime glass and (**b**) FTO coated glass when exposed to 85% RH at 25 °C. RH is introduced at 4 and 5 min for (**a**) and (**b**), respectively.

Figure 4a shows the variation of spectra in *ε* when a MA_0.7_FA_0.3_PbI_3_ film deposited on soda lime glass substrate is exposed to 85% RH at 25 °C. There is a minimal decrease in magnitude of spectra in *ε* compared to MAPbI_3_ deposited on either soda lime glass or FTO coated glass. Mixed MA + FA *A*-cation perovskites have been reported to be more stable against humidity exposure than perovskites with only MA as the *A*-cation [52]. By mixing MA and FA *A*-cations in solid solutions, the cubic perovskite phase is better stabilized. Minimal variation of effective thickness with time for MA_0.7_FA_0.3_PbI_3_ deposited on glass is observed compared to that observed for MAPbI_3_ on glass (Table 1) within a similar time interval. The improved stability of mixed MA + FA perovskite can be attributed to the larger size of the FA cation compared to MA leading to a more favorable tolerance factor [14,53]. The ionic radii of MA and FA are 2.16 and 2.53 Å [54], respectively, and a larger FA cation fraction in the perovskite results in a more ideal Goldschmidt tolerance factor closer to 1 [14,54]. The corresponding tolerance factor values are 0.964 for MA_0.7_FA_0.3_PbI_3_ and 0.911 for MAPbI_3_. The obtained tolerance factor values from calculation are reasonable considering the observed stability of the mixed MA + FA perovskite over MAPbI_3_.

**Figure 5:**
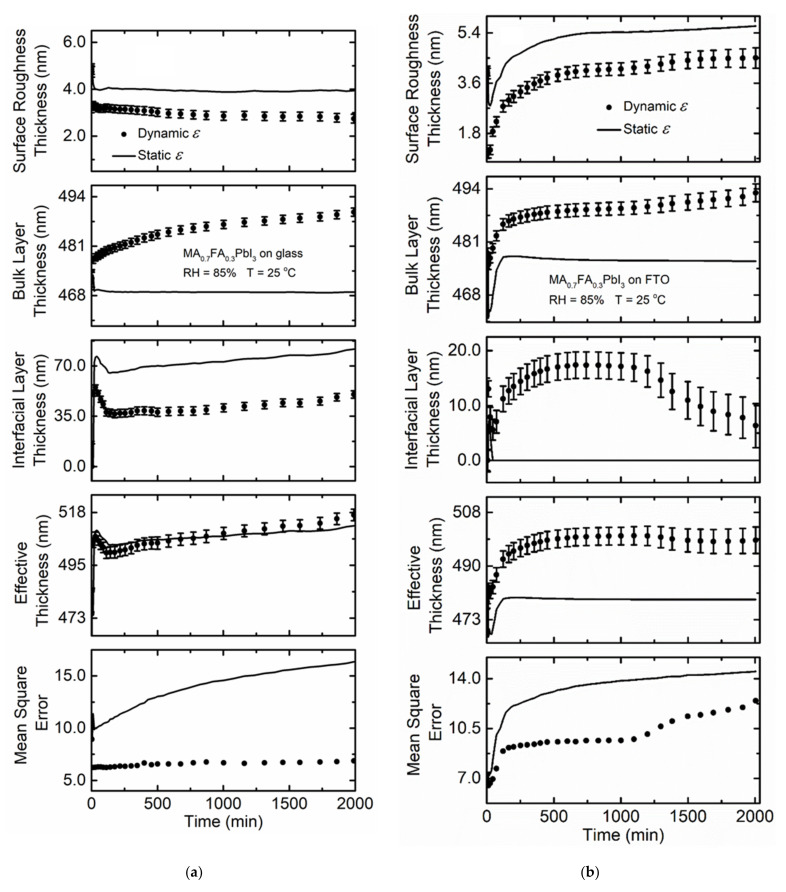
Surface roughness thickness, bulk layer thickness, interfacial layer thickness, effective perovskite thickness, and the quality of fit between the model and experimental ellipsometric spectra in the term of mean square error as functions of time for thin film MA_0.7_FA_0.3_PbI_3_ on (**a**) soda lime glass and (**b**) FTO coated glass when exposed to 85% RH at 25 °C. Values obtained assuming the initial spectra in *ε* (time = 0 min; static) prior to exposure are represented by a solid line, and spectra in *ε* fit at each time point (dynamic) are represented by solid circles. RH is introduced at 4 and 5 min for (**a**) and (**b**), respectively.

The MA_0.7_FA_0.3_PbI_3_ film deposited on FTO coated glass substrate exposed to 85% RH at 25 °C shows a smaller decrease in magnitude of spectra in *ε* (Figure 4b) compared to MA_0.7_FA_0.3_PbI_3_ deposited on soda lime glass. The improved stability over the MA_0.7_FA_0.3_PbI_3_ film deposited on soda lime glass might be due to the influence of the chemical and structural nature of the underlying FTO [51] similar to that observed for MAPbI_3_. Variation in effective thickness is less than that of the film on soda lime glass as shown in Table 1, although both films are relatively stable overall as shown in Figure 5. The *MSE* values for the MA_0.7_FA_0.3_PbI_3_ perovskites on either substrate are low indicating that there are likely no other phases present or changes in phase structure during the observation time. Additionally, the *MSE* values in each case assuming either spectra in *ε* are fixed to the pre-humidity exposure value or allowed to vary with time are very similar when compared to the substantial differences observed for MAPbI_3_.

**Figure 6:**
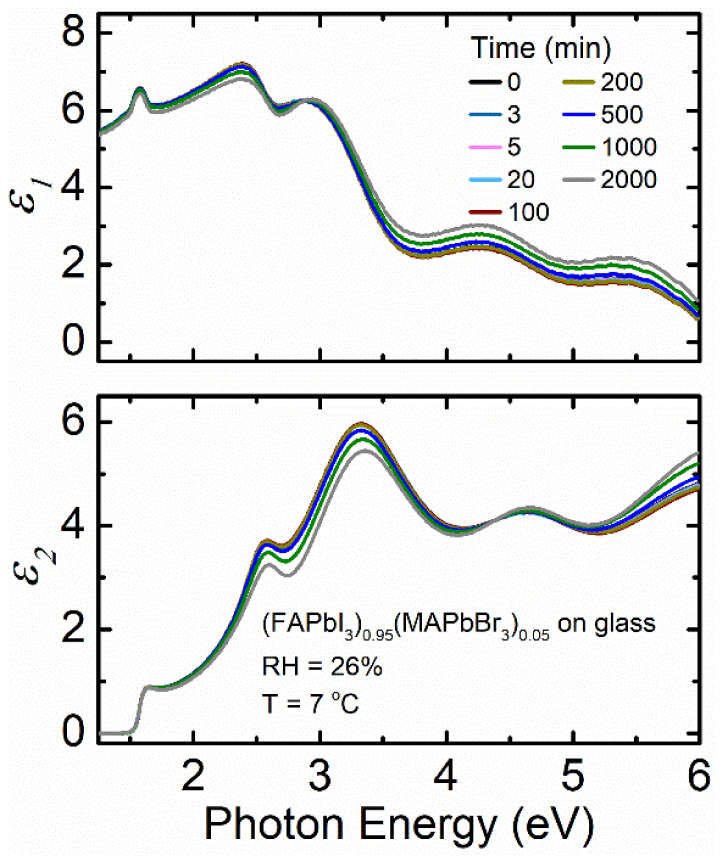
Spectra in *ε* at selected time points for thin film (FAPbI_3_)_0.95_(MAPbBr_3_)_0.05_ deposited on soda lime glass as a function of photon energy when exposed to 26% RH at 7 °C. RH is introduced at 5 min.

To further understand the factors that influence the stability of different perovskite film compositions, (FAPbI_3_)_0.95_(MAPbBr_3_)_0.05_ thin films deposited on soda lime glass substrates have been exposed to 26% RH at 7 °C, 26% RH at 70 °C, and 85% RH at 25 °C. Figure 6 shows spectra in *ε* for (FAPbI_3_)_0.95_(MAPbBr_3_)_0.05_ deposited on soda lime glass exposed to 26% RH at 7 °C. The decrease in magnitude of spectra in *ε* is minimal compared to both MA_0.7_FA_0.3_PbI_3_ and MAPbI_3_.

**Figure 7:**
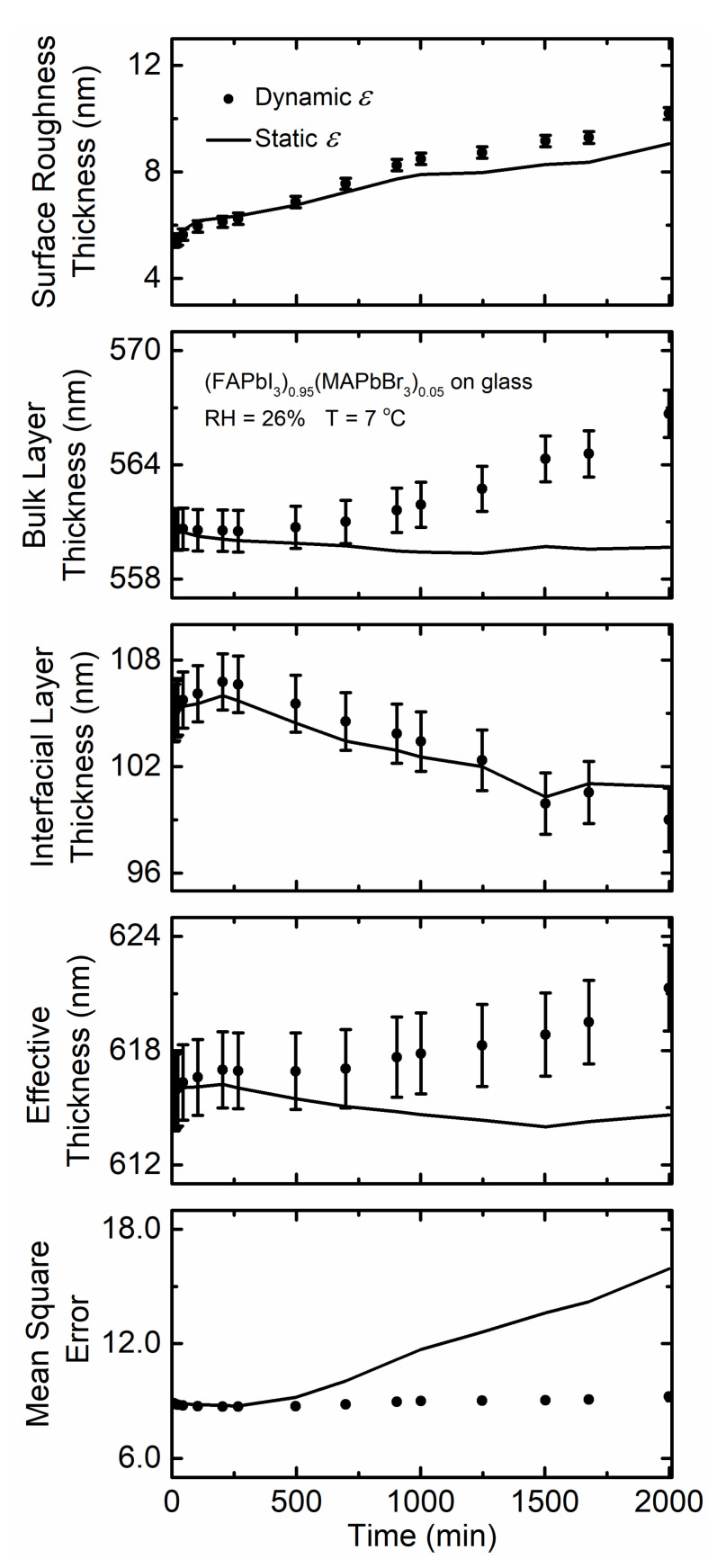
Surface roughness thickness, bulk layer thickness, interfacial layer thickness, effective perovskite thickness, and the quality of fit between the model and experimental ellipsometric spectra in the terms of mean square error as functions of time for (FAPbI_3_)_0.95_(MAPbBr_3_)_0.05_ thin film deposited on soda lime glass when exposed to 26% RH at 7 °C. Values obtained assuming the initial spectra in *ε* (time = 0 min; static) prior to exposure are represented by a solid line, and spectra in *ε* fit at each time point (dynamic) are represented by solid circles. RH is introduced at 5 min.

There is a small variation in effective thickness (Figure 7) when the film is exposed for a prolonged time. Improved quality of fit reflected in the low *MSE* assuming static *ε* obtained prior to humidity exposure and dynamic *ε* are evident, and the gap between the *MSE* describing the two increases only moderately during exposure to humidity. This indicates that low temperature humidity exposure does not affect the stability of this perovskite film substantially, and there are likely no other phases present.

**Figure 8:**
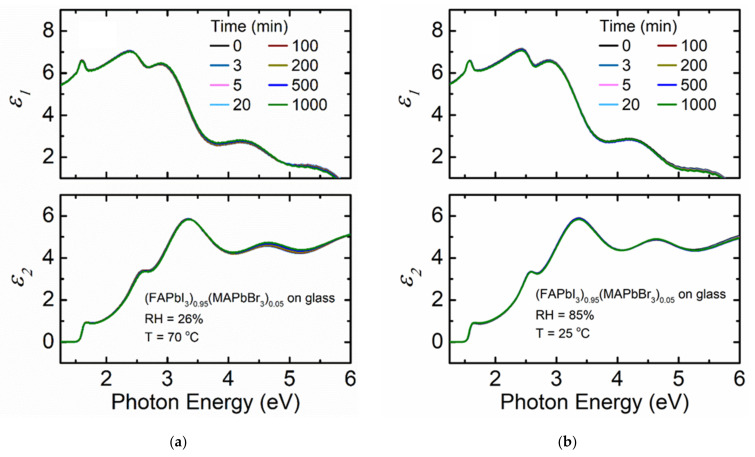
Spectra in *ε* at selected time points for thin film (FAPbI_3_)_0.95_(MAPbBr_3_)_0.05_ deposited on a soda lime glass as a function of photon energy when exposed to (**a**) 26% RH at 70 °C and (**b**) 85% RH at 25 °C. RH is introduced at 5 min.

Figure 8 shows the variation of spectra in *ε* when a (FAPbI_3_)_0.95_(MAPbBr_3_)_0.05_ film deposited on soda lime glass substrate is exposed to the sequence of 26% humidity at 70 °C initially and then 85% RH at 25 °C later. The goal of this combined measurement is to test how this film composition resists varying exposure at both moderate humidity at high temperature and high humidity at moderate temperature after determining its stability at fixed 7 °C and 26% RH conditions. There is no change in magnitude of spectra in *ε* as a function of time compared to MA_0.7_FA_0.3_PbI_3_ and MAPbI_3_, and the changes are even smaller compared to those observed in this composition perovskite at a low temperature. The (FAPbI_3_)_0.95_(MAPbBr_3_)_0.05_ film at a low temperature may exhibit a small change over time as small amounts of water condense onto the film. However, even those changes are minor compared to the other perovskite compositions studied.

**Figure 9:**
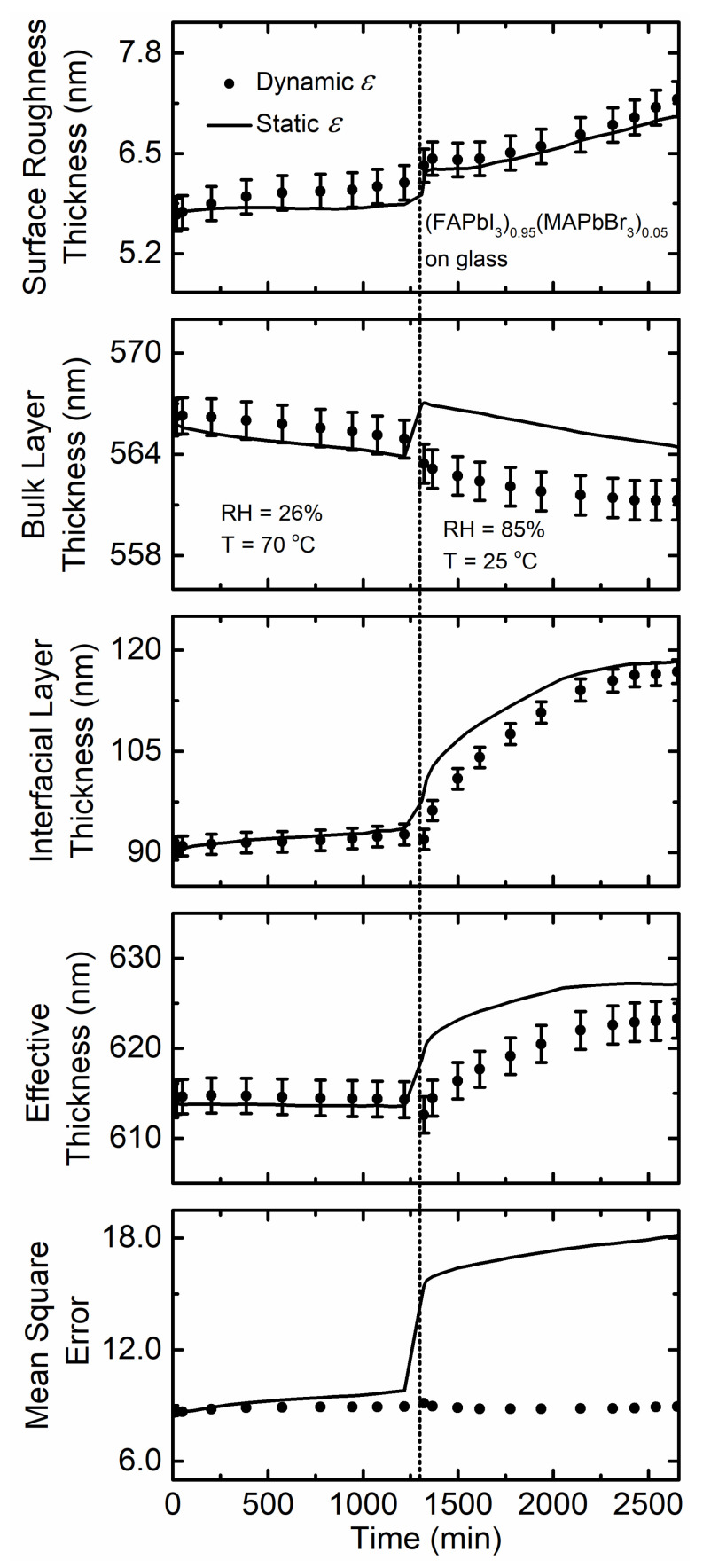
Surface roughness thickness, bulk layer thickness, interfacial layer thickness, effective perovskite thickness, and the quality of fit between the model and experimental ellipsometric spectra in the term of mean square error as functions of time for thin film (FAPbI_3_)_0.95_(MAPbBr_3_)_0.05_ deposited on soda lime glass when exposed to 26% RH at 70 °C then 85% RH at 25 °C. Values obtained assuming the initial spectra in *ε* (time = 0 min; static) prior to exposure are represented by a solid line, and spectra in *ε* fit at each time point (dynamic) are represented by solid circles. The vertical dotted line marks when the temperature and humidity are changed. RH is introduced at 5 min.

Figure 9 illustrates the structural parameters in the form of the surface roughness, bulk layer, and interfacial layer thicknesses as functions of time for the (FAPbI_3_)_0.95_(MAPbBr_3_)_0.05_ film deposited on soda lime glass substrate exposed to 26% RH at 70 °C then 85% RH at 25 °C. No variation in effective thickness is observed for the film exposed to 26% RH at 70 °C but there is a minimal increase in effective thickness during 85% RH at 25 °C exposure in the reported time interval in Table 1. This variation is minimal compared to the other film compositions and measurement conditions. The small changes that are present (<10 nm) are attributed to small precession of the measurement beam spot on the sample surface over the time of the measurement. The quality of fit assuming a static *ε* obtained prior to humidity exposure is almost identical to that when spectra in *ε* are obtained at each time point (dynamic *ε*) for the time exposed to 26% RH at 70 °C. Note that the same static *ε* obtained prior to humidity exposure at 70 °C is used as the temperature is decreased, leading to a small increase in the *MSE*. This difference is expected as spectra in *ε* for semiconductors will vary with measurement temperature [55,56,57]. However, even in this case the spectra in *ε* measured at 70 °C provides a low *MSE*. This similarity in quality of fit indicates that the structural parameters of the film and the intrinsic characteristics of this perovskite reflected in *ε* are not affected by humidity exposure and temperature variations. Overall, temperature variations over the 7 to 70 °C ranges do not seem to have an impact on the stability of the (FAPbI_3_)_0.95_(MAPbBr_3_)_0.05_ composition perovskite films.

The improved stability for (FAPbI_3_)_0.95_(MAPbBr_3_)_0.05_ over MAPbI_3_ and MA_0.7_FA_0.3_PbI_3_ with temperature variations and humidity exposure is attributed to the incorporation of Br into the mixed MA + FA film. Previous research has shown that including a small amount of Br enhances the stability, suppresses ion migration, and reduces trap-state density [53]. According to Noh et al., upon incorporation of Br into MAPbI_3_ for solar cell absorbers, there is an improvement in open circuit voltage (V_OC_) from 0.87 to 1.13 V and an increase in fill factor (FF) from 0.66 to 0.74 [58]. Increases in these parameters is an indicator of good electronic film quality [36]. It has also been reported that replacing larger I ions with smaller Br ions results in a greater resistance to high humidity at room temperature [53]. Furthermore, perovskite films with mixed cations and mixed anions crystallize at much higher temperatures and exhibit greater degrees of crystalline order. A material with enhanced crystallinity both can promote high V_OC_ in PV devices and are more stable against degradation [4]. The two step-preparation solution-processing method for (FAPbI_3_)_0.95_(MAPbBr_3_)_0.05_ also leads to larger grain size than in the single step approach which further enhances film stability [33].

Bandgap energies of the studied perovskites films (Table 1) are not substantially affected by prolonged exposure to temperature and humidity, although there is observed variation in the magnitude of spectra in *ε* above the bandgap. There is a slight decrease in bandgap when the (FAPbI_3_)_0.95_(MAPbBr_3_)_0.05_ film exposed to 26% RH at 70 °C is cooled to 85% RH at 25 °C. Previous research has shown that the bandgap decreases with decreasing temperature in these hybrid halide perovskites unlike in other semiconductors where the bandgap decreases with increasing temperature as a result of lattice dilatation [55,57]. Here, a minimal decrease in bandgap is observed with decreased temperature. This change is small on its own since the temperature variation is not extreme. Film composition also controls variation in bandgap [59]. Lack of variation in bandgap in the case of the perovskite films continuously exposed to a fixed temperature and humidity can be attributed to the fact that the film composition remains largely the same during the time of exposure at this range of temperatures. Even MAPbI_3_, which exhibits substantial degradation by water molecules, retains a similar bandgap energy throughout humidity exposure although the higher energy CPs and magnitude of *ε* change more substantially.

## 4. Conclusions

The stability of optical and structural properties of organic–inorganic metal halides-based perovskites thin films exposed to various humidity and temperature conditions have been explored using RTSE. MAPbI_3_ degrades upon exposure to 85% RH at room temperature whereas mixed MA + FA perovskites shows improved stability when exposed to RH at various temperatures. Temperature alone in the range of 7 to 70 °C does not affect the stability of studied (FAPbI_3_)_0.95_(MAPbBr_3_)_0.05_ perovskite films. There is an influence of the substrate on the stability of these perovskites as the films deposited on FTO coated glass shows enhanced stability compared to the films deposited directly onto soda lime glass. Additionally, incorporation of bromine and the two-step preparation method are among other factors for improved stability of the mixed MA + FA cation perovskites. Among the studied perovskites, (FAPbI_3_)_0.95_(MAPbBr_3_)_0.05_ demonstrated a robust stability for the tested humidity and temperature conditions.

## Figures and Tables

**Table 1:** *MSE* range; initial effective thickness; effective thickness change; and minimum, maximum, and average bandgap values for perovskites films over 0 to 550 min of exposure time as obtained from time dependent fitting of spectra in *ε*.

Perovskite Films	*MSE* Range	Initial Effective Thickness (nm)	Effective Thickness Change (nm)	Minimum Bandgap (eV)	Maximum Bandgap (eV)	Average Bandgap (eV)
MAPbI_3_ on glass	10.6–35.5	486.8	218	1.596	1.605	1.604
MAPbI_3_ on FTO	13.5–14.5	516.9	23	1.600	1.613	1.605
MA_0.7_FA_0.3_PbI_3_ on glass	6.2–6.5	474.3	16	1.590	1.593	1.590
MA_0.7_FA_0.3_PbI_3_ on FTO	6.7–9.6	478.1	12	1.606	1.616	1.613
(FAPbI_3_)_0.95_(MAPbBr_3_)_0.05_ on glass at 7 °C with 26% RH	8.7–8.8	615.8	1	1.560	1.565	1.562
(FAPbI_3_)_0.95_(MAPbBr_3_)_0.05_ on glass at 70 °C with 26% RH	8.6–8.9	614.2	0	1.590	1.595	1.593
(FAPbI_3_)_0.95_(MAPbBr_3_)_0.05_ on glass at 25 °C with 85% RH	8.9–8.8	614.5	7	1.559	1.571	1.563

## Data Availability

The data presented in this study are available on request from the corresponding author.
